# Witnessing Community Violence, Gun Carrying, and Associations with Substance Use and Suicide Risk Among High School Students — Youth Risk Behavior Survey, United States, 2021

**DOI:** 10.15585/mmwr.su7201a3

**Published:** 2023-04-28

**Authors:** Christopher R. Harper, Jingjing Li, Kameron Sheats, Marci F. Hertz, Molly Merrill-Francis, Norah W. Friar, Carmen L. Ashley, Shari Shanklin, Colleen Barbero, Elizabeth M. Gaylor, Brooke E. Hoots

**Affiliations:** ^1^Division of Violence Prevention, National Center for Injury Prevention and Control, CDC; ^2^Division of Adolescent and School Health, National Center for HIV, Viral Hepatitis, STD, and TB Prevention, CDC; ^3^Division of Overdose Prevention, National Center for Injury Prevention and Control, CDC; ^4^Division of Injury Prevention, National Center for Injury Prevention and Control, CDC

## Abstract

Community violence, including homicides involving firearms, is a significant public health concern. From 2019 to 2020, firearm-related homicides increased by 39% for youths and young adults aged 10–24 years, and rates of suicide by firearm increased by approximately 15% among the same age group. Findings from the nationally representative 2021 Youth Risk Behavior Survey were used to analyze disparities and correlates of witnessing community violence and gun carrying among a nationally representative sample of high school students. Chi-square tests and logistic regression accounting for the complex sampling of the survey were used to assess demographic differences by student sex, race and ethnicity, age, and sexual identity in ever witnessing community violence, gun carrying in the past 12 months, and their associations with substance use and suicide risk. Measures of substance use included current binge drinking and marijuana use and lifetime prescription opioid misuse and illicit drug use. Suicide risk included seriously considered attempting suicide and attempted suicide in the past 12 months. Overall, approximately 20% of students witnessed community violence and 3.5% of students carried a gun. American Indian or Alaska Native, Black, and Hispanic students were more likely to witness community violence and to report carrying a gun than their White peers. Males were more likely to witness community violence and carry a gun than females. Lesbian, gay, or bisexual students were more likely to witness community violence than their heterosexual peers. Also, witnessing community violence consistently was associated with increased odds of gun carrying, substance use, and suicide risk for both males and females and when comparing Black, White, and Hispanic students. These findings highlight the importance of comprehensive violence prevention strategies that incorporate health equity to mitigate the effects of violence exposure on substance use and suicide risk among youths.

## Introduction

Community violence is defined as violence between unrelated persons who might or might not know each other, generally outside the home (https://www.cdc.gov/violenceprevention/communityviolence/index.html). From 2019 to 2020, firearm-related homicides, including community violence, increased by 39% for youths and young adults aged 10–24 years, with rates of suicide by firearm increasing by 15% in the same age group (*1*). In 2020, firearm-related injuries caused more deaths of persons aged 1–19 years than any other injury or other cause of death (*2*). Exposure to violence has serious health consequences across a person’s lifespan. Witnessing community violence and firearm carrying have both been linked to increased substance use and suicide risk among youths (*3*–*5*). The longitudinal Project on Human Development in Chicago Neighborhoods found that among children and adolescents aged 9–15 years, witnessing community violence was associated with alcohol use, smoking, and marijuana use, in addition to suicide risk (*3*). Exposure to community violence also might increase risk for violence perpetration. Youths who either commit or experience different forms of violence are at higher risk for perpetrating violence later in adolescence and in adulthood, and exposure to community violence is a risk factor for gun carrying (*3*,*4*).

Different communities, populations, and racial and ethnic groups face disproportionate exposure to community violence related to structural racism and inequities that might have increased during the COVID-19 pandemic (*1*). For example, the rate of homicides by firearm among Black or African American (Black) males aged 10–24 years was 20.6 times as high as that among White males of the same age in 2019, and this ratio increased to 21.6 in 2020 (*1*). Data from the 2021 Youth Risk Behavior Survey (YRBS) were analyzed to better understand disparities and correlates of witnessing community violence and gun carrying, including differences in the prevalence of witnessing community violence and gun carrying by sex, race and ethnicity, age, and sexual identity and associations among witnessing community violence, gun carrying, suicide risk, and substance use by sex and race and ethnicity. This is the first report using nationally representative YRBS data to examine the associations between witnessing community violence and gun carrying. Findings might be used to develop community- and school-based strategies to prevent violence and mitigate the effects of violence exposure and gun carrying on youths at disproportionate risk for violence victimization and perpetration.

## Methods

### Data Source

This report includes data from the 2021 YRBS (N = 17,232), a cross-sectional, school-based survey conducted biennially since 1991. Each survey year, CDC collects data from a nationally representative sample of public and private school students in grades 9–12 in the 50 U.S. states and the District of Columbia. Additional information about YRBS sampling, data collection, response rates, and processing is available in the overview report of this supplement (*6*). The prevalence estimates for witnessing community violence and gun carrying for the overall study population and by sex, race and ethnicity, grade, and sexual identity are available at https://nccd.cdc.gov/youthonline/App/Default.aspx. The full YRBS questionnaire, data sets, and documentation are available at https://www.cdc.gov/healthyyouth/data/yrbs/index.htm. This activity was reviewed by CDC and was conducted consistent with applicable federal law and CDC policy.*

### Measures

The primary health risk behaviors examined were ever witnessing community violence and past-year gun carrying. The analysis included two measures of suicide risk (seriously considered attempting suicide and attempted suicide in the past 12 months) and four measures of substance use (current binge drinking, current marijuana use, lifetime prescription opioid misuse, and lifetime illicit substance use). All variables were binary and coded with the absence of the behavior or exposure as the reference category (Table 1). Demographic variables included sex (female and male), sexual identity (heterosexual, lesbian, gay, bisexual, questioning, or other), and race and ethnicity (American Indian or Alaska Native [AI/AN], Asian, Black, Native Hawaiian or other Pacific Islander, White, Hispanic or Latino [Hispanic], and multiracial). (Persons of Hispanic origin might be of any race but are categorized as Hispanic; all racial groups are non-Hispanic.) Age was categorized into three groups for ease of comparison (≤15 years, 16–17 years, and ≥18 years).

**TABLE 1:** Health risk behavior measures — Youth Risk Behavior Survey, United States, 2021

Behavior	Question	Response option	Analytic coding
**Violence-related outcomes**			
Witnessed community violence	Have you ever seen someone get physically attacked, beaten, stabbed, or shot in your neighborhood?	Yes or no	Yes versus no
Gun carrying	During the past 12 months, on how many days did you carry a gun?	0 days; 1 day; 2 or 3 days; 4 or 5 days; or ≥6 days	≥1 day versus 0 days
**Suicide risk**			
Seriously considered attempting suicide	During the past 12 months, did you ever seriously consider attempting suicide?	Yes or no	Yes versus no
Attempted suicide	During the past 12 months, how many times did you actually attempt suicide?	0 times; 1 time; 2 or 3 times; 4 or 5 times; or ≥6 times	≥1 time versus 0 times
**Substance use**			
Current binge drinking	During the past 30 days, on how many days did you have 4 or more drinks of alcohol in a row, that is, within a couple of hours (if you are female) or 5 or more drinks of alcohol in a row, that is, within a couple of hours (if you are male)?	0 days; 1 day; 2 days; 3–5 days; 6–9 days; 10–19 days; or ≥20 days	≥1 day versus 0 days
Current marijuana use	During the past 30 days, how many times did you use marijuana?	0 times; 1 or 2 times; 3–9 times; 10–19 times; 20–39 times; or ≥40 times	≥1 time versus 0 times
Lifetime prescription opioid misuse	The next 2 questions ask about the use of prescription pain medicine without a doctor’s prescription or differently than how a doctor told you to use it. For these questions, count drugs such as codeine, Vicodin, OxyContin, Hydrocodone, and Percocet. During your life, how many times have you taken prescription pain medicine without a doctor’s prescription or differently than how a doctor told you to use it?	0 times; 1 or 2 times; 3–9 times; 10–19 times; 20–39 times; or ≥40 times	≥1 time versus 0 times
Lifetime illicit drug use	Calculated variable based upon responses to the following questions: heroin, cocaine, methamphetamines, synthetic marijuana, ecstasy, hallucinogenic drugs, and inhalants. • During your life, how many times have you sniffed glue, breathed the contents of aerosol spray cans, or inhaled any paints or sprays to get high? • During your life, how many times have you used synthetic marijuana? • During your life, how many times have you used any form of cocaine, including powder, crack, or freebase? • During your life, how many times have you used heroin (also called smack, junk, or China white)? • During your life, how many times have you used methamphetamines (also called speed, crystal meth, crank, ice, or meth)? • During your life, how many times have you used ecstasy (also called MDMA or Molly)? • During your life, how many times have you used hallucinogenic drugs, such as LSD, acid, PCP, angel dust, mescaline, or mushrooms?	0 times; 1 or 2 times; 3–9 times; 10–19 times; 20–39 times; or ≥40 times	≥1 time for at least 1 of the included questions versus 0 times for all included questions

### Analysis

Descriptive analyses were conducted to determine the point prevalence estimates and corresponding 95% CIs for ever witnessing community violence and gun carrying in the past 12 months in overall samples and by sex (male versus female) and by the three largest racial and ethnic groups (Black, White, and Hispanic) because of sample size constraints. Chi-square tests and *t*-tests with Taylor series linearization were used to compare demographic group differences. Associations between witnessing community violence and independent variables (gun carrying, suicide risk, and substance use) were assessed in separate sex- or race and ethnicity–stratified adjusted logistic regression models, which generated adjusted prevalence ratios and corresponding 95% CIs for each independent variable. Associations between gun carrying and independent variables (suicide risk and substance use) were assessed in nonstratified adjusted models. All regression models were controlled for sex, age, race and ethnicity, and sexual identity. Estimates were considered statistically significant if the 95% CI did not include 1.0, p value was <0.05, or both. All analyses were conducted in SAS-callable SUDAAN (version 11.0.3; RTI International) using sample weights to account for complex survey design and nonresponse (*6*).

## Results

Overall, 19.9% of high school students reported ever witnessing community violence, and 3.5% reported carrying a gun during the previous 12 months. Ever witnessing community violence and gun carrying were more prevalent among males than females and for AI/AN, Black, Hispanic, and multiracial students than for Asian or White students (Table 2). Gun carrying during the past 12 months was significantly more prevalent among students aged ≥18 years compared with students aged ≤15 years. However, no statistically significant differences existed in witnessing community violence by age. Lesbian, gay, or bisexual students were more likely to witness community violence than their heterosexual peers; however, differences in gun carrying by sexual identity were not statistically significant.

**TABLE 2:** Witnessing community violence and gun carrying, by student characteristics — Youth Risk Behavior Survey, United States, 2021*

Characteristic	Witnessing community violence			Gun carrying		
	Yes	No	Chi-square test p value^†^	Yes	No	Chi-square test p value^†^
	% (95%CI)	% (95%CI)		% (95% CI)	% (95% CI)	
**Overall**	19.9 (17.3–22.7)	80.1 (77.3–82.7)	NA	3.5 (2.8–4.4)	96.5 (95.6–97.2)	NA
**Sex**	NA	NA	0.024	NA	NA	0.000
Female	19.2 (16.6–22.1)	80.8 (77.9–83.4)	NA	1.8 (1.5–2.3)	98.2 (97.7–98.5)	NA
Male	20.4 (17.8–23.2)	79.6 (76.8–82.2)	NA	5.0 (3.9–6.3)	95.0 (93.7–96.1)	NA
**Race and ethnicity^§^**	NA	NA	0.000	NA	NA	0.000
American Indian or Alaska Native	26.0 (18.6–35.1)^¶,^**	74.0 (64.9–81.4)	NA	5.3 (2.5–11.1)**	94.7 (88.9–97.5)	NA
Asian	9.3 (6.7–12.8)^¶,††,§§,¶¶,***^	90.7 (87.2–93.3)	NA	1.0 (0.5–1.8)^¶,††,§§,¶¶,^***	99.0 (98.2–99.5)	NA
Black or African American	29.3 (25.8–33.2)^¶^	70.7 (66.8–74.2)	NA	5.1 (4.2–6.3)*^¶^*^,¶¶,^***	94.9 (93.7–95.8)	NA
Native Hawaiian or other Pacific Islander	26.2 (22.0–30.9)	73.8 (69.1–78.0)	NA	3.9 (2.9–5.3)	96.1 (94.7–97.1)	NA
White	24.5 (19.1–30.8)^¶¶,^***	75.5 (69.2–80.9)	NA	3.0 (1.6–5.5)	97.0 (94.5–98.4)	NA
Hispanic or Latino	21.3 (11.8–35.4)	78.7 (64.6–88.2)	NA	5.1 (0.7–29.6)	94.9 (70.4–99.3)	NA
Multiracial	14.8 (12.9–17.0)	85.2 (83.0–87.1)	NA	3.0 (2.4–3.8)	97.0 (96.2–97.6)	NA
**Age group, yrs**	NA	NA	0.281	NA	NA	0.046
≤15	18.8 (16.8–21.0)	81.2 (79.0–83.2)	NA	3.2 (2.5–4.1)	96.8 (95.9–97.5)	NA
16–17	20.5 (17.0–24.5)	79.5 (75.6–83.0)	NA	3.5 (2.6–4.6)	96.5 (95.4–97.4)	NA
≥18	22.5 (17.4–28.6)	77.5 (71.4–82.6)	NA	6.5 (4.2–9.9)^†††^	93.5 (90.1–95.8)	NA
**Sexual identity**						
Lesbian, gay, or bisexual	27.0 (23.9–30.3)^§§§,¶¶¶^	73.0 (69.7–76.1)	0.000	2.9 (1.9–4.3)	97.1 (95.7–98.1)	0.287
Heterosexual	18.2 (15.7–20.9)	81.8 (79.1–84.3)	NA	3.3 (2.7–4.1)	96.7 (95.9–97.3)	NA
Questioning or other	20.4 (17.7–23.4)	79.6 (76.6–82.3)	NA	4.6 (2.9–7.2)	95.4 (92.8–97.1)	NA

**Abbreviation:** NA = not applicable.

* N = 17,232 respondents. Because the state and local questionnaires differ by jurisdiction, students in these schools were not asked all national YRBS questions. Therefore, the total number (N) of students answering each question varied. Percentages in each category are calculated on the known data.

^†^ Chi-square tests were applied to examine the bivariate relationships between demographic characteristics and witnessing community violence or gun carrying. Statistical significance is defined as p<0.05 for the chi-square test.

^§ ^Persons of Hispanic or Latino (Hispanic) origin might be of any race but are categorized as Hispanic; all racial groups are non-Hispanic.

^¶^ Significantly different from White students, on the basis of *t*-test analysis with Taylor series linearization (p<0.05).

** Significantly different from Asian students, on the basis of *t*-test analysis with Taylor series linearization (p<0.05).

^††^ Significant difference from Black or African American students, on the basis of *t*-test analysis with Taylor series linearization (p<0.05).

^§§^ Significantly different from American Indian or Alaska Native students, on the basis of *t*-test analysis with Taylor series linearization (p<0.05).

^¶¶^ Significantly different from multiracial students, on the basis of *t*-test analysis with Taylor series linearization (p<0.05).

*** Significantly different from Hispanic students, on the basis of *t*-test analysis with Taylor series linearization (p<0.05).

^†††^ Significantly different from students aged ≤15 years, on the basis of *t*-test analysis with Taylor series linearization (p<0.05).

^§§§^ Significantly different from heterosexual students, on the basis of *t*-test analysis with Taylor series linearization (p<0.05).

^¶¶¶^ Significantly different from questioning or other students, on the basis of *t*-test analysis with Taylor series linearization (p<0.05).

Witnessing community violence was more prevalent among students who carried a gun, and suicide risk and substance use also were associated with witnessing community violence (Tables 3 and 4). Suicide risk and substance use were associated with gun carrying (Table 5).

**TABLE 3:** Prevalence of witnessing community violence among high school students, by gun carrying, suicide risk, and substance use behaviors and experiences and sex — Youth Risk Behavior Survey, United States, 2021*

Behavior/Experience	Witnessed community violence					
	Male			Female		
	Did not experience the risk behavior % (95% CI)	Experienced the risk behavior % (95% CI)	aPR^†,§^ (95% CI)	Did not experience the risk behavior % (95% CI)	Experienced the risk behavior % (95% CI)	aPR^†,§^ (95% CI)
**Gun carrying^¶^**	18.9 (16.5–21.4)	56.7 (49.2–63.8)	3.1 (2.7–3.5)	18.7 (16.1–21.7)	61.2 (50.9–70.6)	3.0 (2.4–3.8)
**Suicide risk**						
Seriously considered attempting suicide^¶^	17.5 (15.2–20.2)	37.1 (31.4–43.3)	2.1 (1.8–2.5)	13.8 (10.4–17.9)	32.1 (27.6–36.9)	2.2 (1.6–3.0)
Attempted suicide^¶^	18.1 (15.7–20.9)	44.5 (38.0–51.3)	2.3 (1.9–2.7)	15.3 (13.1–18.0)	42.9 (38.7–47.2)	2.5 (2.2–2.9)
**Substance use**						
Current binge drinking**	18.3 (15.8–21.1)	34.2 (27.0–42.2)	1.9 (1.5–2.4)	17.0 (14.5–19.8)	32.0 (25.4–39.4)	1.9 (1.6–2.3)
Current marijuana use^††^	16.9 (14.7–19.4)	41.0 (35.1–47.1)	2.3 (2.0–2.7)	14.7 (12.3–17.4)	39.1 (35.3–43.1)	2.4 (2.0–2.9)
Lifetime prescription opioid misuse	18.4 (16.2–20.9)	38.1 (30.6–46.1)	2.0 (1.7–2.4)	15.7 (13.4–18.3)	39.0 (33.5–44.9)	2.3 (1.9–2.8)
Lifetime illicit drug use^§§^	18.0 (15.5–20.9)	39.8 (31.9–48.4)	2.2 (1.9–2.5)	15.8 (13.3–18.7)	41.1 (36.1–46.3)	2.5 (2.1–2.9)

**Abbreviation:** aPR = adjusted prevalence ratio.

* N = 17,232 respondents. Because the state and local questionnaires differ by jurisdiction, students in these schools were not asked all national YRBS questions. Therefore, the total number (N) of students answering each question varied. Percentages in each category are calculated on the known data.

^†^ aPRs were estimated with gun carrying, suicide risk, and substance use variables as the outcome.

^§^ Logistic models adjusted for age, race and ethnicity, and sexual identity. Estimates were considered statistically significant if the 95% CIs did not include 1.0.

^¶^ During the 12 months before the survey

** Had four or more drinks of alcohol in a row (if they were female) or five or more drinks of alcohol in a row (if they were male) within a couple of hours on ≥1 day during the 30 days before the survey.

^††^ One or more times during the 30 days before the survey.

^§§^ Lifetime use of at least one of the following: cocaine, ecstasy, hallucinogenic drugs, heroin, inhalants, methamphetamines, or synthetic marijuana.

**TABLE 4:** Prevalence of witnessing community violence among high school students, by gun carrying, suicide risk, and substance use behaviors and experiences and race and ethnicity* — Youth Risk Behavior Survey, United States, 2021^†^

Behavior/Experience	Witnessed community violence								
	Black or African American			White			Hispanic or Latino		
	Did not experience the risk behavior % (95% CI)	Experienced the risk behavior % (95% CI)	aPR^§,¶^ (95% CI)	Did not experience the risk behavior % (95% CI)	Experienced the risk behavior % (95% CI)	aPR^§,¶^ (95% CI)	Did not experience the risk behavior % (95% CI)	Experienced the risk behavior % (95% CI)	aPR^§,¶^ (95% CI)
Gun carrying**	26.9 (23.5–30.7)	70.8 (54.8–83.0)	2.6 (2.1–3.2)	14.2 (12.4–16.3)	45.6 (33.6–58.2)	3.6 (2.9–4.3)	24.6 (20.5–29.2)	64.4 (55.0–72.8)	2.7 (2.3–3.3)
**Suicide risk**									
Seriously considered attempting suicide	26.1 (22.1–30.4)	41.6 (34.2–49.5)	1.7 (1.2–2.3)	10.6 (8.9–12.5)	29.4 (26.2–32.8)	2.8 (2.5–3.2)	22.3 (16.8–28.9)	40.5 (32.6–48.8)	1.8 (1.3–2.6)
Attempted suicide**	28.0 (23.9–32.7)	45.3 (38.7–52.1)	1.6 (1.3–2.0)	12.3 (10.5–14.3)	40.7 (34.3–46.7)	3.1 (2.7–3.6)	23.4 (19.5–27.8)	45.5 (36.0–55.4)	2.0 (1.7–2.3)
**Substance use**									
Current binge drinking^††^	27.1 (23.4–31.1)	58.8 (39.1–76.1)	2.1 (1.5–2.9)	12.8 (11.1–14.8)	26.1 (19.5–34.0)	2.0 (1.6–2.5)	22.8 (18.7–27.5)	43.5 (36.8–50.4)	1.9 (1.5–2.5)
Current marijuana use^§§^	24.2 (20.5–28.4)	47.0 (39.2–55.1)	1.9 (1.5–2.4)	11.7 (9.9–13.7)	32.3 (27.9–37.0)	2.8 (2.2–3.4)	22.0 (18.9–25.6)	46.8 (39.8–53.8)	2.1 (1.8–2.3)
Lifetime prescription opioid misuse	26.7 (22.8–31.0)	45.3 (35.3–55.7)	1.7 (1.4–2.2)	12.2 (10.8–13.8)	35.1 (27.5–43.6)	2.8 (2.2–3.6)	23.1 (19.5–27.1)	44.3 (37.9–50.8)	1.9 (1.7–2.2)
Lifetime illicit drug use^¶¶^	29.2 (25.0–33.9)	51.7 (36.5–66.6)	1.7 (1.1–2.6)	11.9 (10.0–14.0)	35.1 (29.5–41.1)	2.9 (2.4–3.4)	23.2 (19.5–27.3)	46.9 (38.0–55.9)	2.0 (1.7–2.3)

**Abbreviation:** aPR = adjusted prevalence ratio.

* Persons of Hispanic or Latino (Hispanic) origin might be of any race but are categorized as Hispanic; all racial groups are non-Hispanic.

^†^ N = 17,232 respondents. Because the state and local questionnaires differ by jurisdiction, students in these schools were not asked all national YRBS questions. Therefore, the total number (N) of students answering each question varied. Percentages in each category are calculated on the known data.

^§^ aPRs were estimated with gun carrying, suicide risk, and substance use variables as the outcome. All aPRs were statistically significant if p<0.05.

^¶^ Logistic models adjusted for age, sex, and sexual identity. Estimates were considered statistically significant if the 95% CIs did not include 1.0.

** During the 12 months before the survey

^††^ Had four or more drinks of alcohol in a row (if they were female) or five or more drinks of alcohol in a row (if they were male) within a couple of hours on ≥1 day during the 30 days before the survey.

^§§^ One or more times during the 30 days before the survey.

^¶¶^ Lifetime use of at least one of the following: cocaine, ecstasy, hallucinogenic drugs, heroin, inhalants, methamphetamines, or synthetic marijuana.

**TABLE 5:** Adjusted prevalence ratios for suicide risk and substance use behavior, by gun carrying — Youth Risk Behavior Survey, United States, 2021*

Risk/Behavior	No, gun carrying % (95% CI)	Yes, gun carrying % (95% CI)	aPR^†^ (95% CI)
**Suicide risk**			
Seriously considered attempting suicide^§^	21.7 (20.7–22.9)	40.2 (35.2–45.5)	2.0 (1.8–2.3)
Attempted suicide^§^	9.3 (8.5–10.1)	36.4 (30.1–43.2)	3.7 (3.1–4.5)
**Substance use**			
Current binge drinking^¶^	9.7 (8.7–10.9)	38.2 (30.5–46.7)	3.9 (3.1–4.8)
Current marijuana use**	14.8 (13.3–16.4)	51.2 (42.8–59.5)	3.3 (2.8–3.9)
Lifetime prescription drug misuse	11.2 (10.4–12.1)	43.5 (36.5–50.9)	4.0 (3.2–5.0)
Lifetime illicit drug use^††^	12.1 (11.1–13.2)	46.9 (41.6–52.2)	3.8 (3.2–4.5)

**Abbreviation:** aPR = adjusted prevalence ratio.

* N = 17,232 respondents. Because the state and local questionnaires differ by jurisdiction, students in these schools were not asked all national YRBS questions. Therefore, the total number (N) of students answering each question varied. Percentages in each category are calculated on the known data.

^†^ Logistic models adjusted for sex, race and ethnicity, age, and sexual identity. Estimates were considered statistically significant if the 95% CI did not include 1.0.

^§^ During the 12 months before the survey.

^¶^ Had four or more drinks of alcohol in a row (if they were female) or five or more drinks of alcohol in a row (if they were male) within a couple of hours on ≥1 day during the 30 days before the survey.

** One or more times during the 30 days before the survey.

^††^ Lifetime use of at least one of the following: cocaine, ecstasy, hallucinogenic drugs, heroin, inhalants, methamphetamines, or synthetic marijuana.

## Discussion

Approximately one in five high school students ever witnessed community violence, and 3.5% of high school students carried a gun during the previous 12 months. Witnessing community violence and gun carrying were associated with student substance use and suicide risk. These findings were consistent with other studies indicating associations between witnessing community violence and gun carrying and increased risk for suicide, substance use, and other adverse health outcomes (*3*,*4*).

Community violence has been described as an adverse childhood experience (ACE), and both ACE exposure and witnessing community violence have been associated with weapon carrying (*5*). Previous research has demonstrated that young persons might carry weapons for self-defense to protect against future violence, particularly when they have been directly victimized or perceive high levels of community violence (*5*). The overall prevalence of witnessing community violence and gun carrying, as well as the statistically significant differences by race and ethnicity and sex highlight the need to implement comprehensive evidence-based prevention strategies in locations that are disproportionately affected by violence.

Findings from the 2021 YRBS indicate that students from most racial and ethnic minority groups were more likely to witness community violence and to report gun carrying than their White peers. The differential exposure by race and ethnicity might increase disparities in other types of morbidity and mortality from substance use or other health outcomes (e.g., chronic disease) because of stress and adversity. Racial and ethnic minorities experience higher rates of violence, which have been explained by discrimination and racism, concentrated poverty, high crime rates, and economic or residential instability (*7*).

Furthermore, findings revealed a substantially higher prevalence of community violence exposure among students who carried a gun compared with those who did not. Gun carrying might be associated with experiences of racism, discrimination, feeling the need to protect oneself because of increased exposure to community violence, mistrust in the criminal justice and other government systems, and poor or inadequate community-level protective factors (*5*). Results also showed differences in exposure to community violence for youths who identified as lesbian, gay, or bisexual. These youths were more likely to witness community violence than those identifying as heterosexual. Sexual minority youths have been found to be at greater risk for substance use, suicide risk, and victimization (*8*). These factors might create an environment where sexual minority students are more likely to witness interpersonal violence because they often are the victim (*9*).

This report also found important associations between witnessing community violence, substance use, and suicide risk. Youths who witnessed community violence were more likely to report carrying a gun, considering or attempting suicide, and engaging in current and lifetime substance use behavior compared with youths who had not witnessed it. Witnessing community violence, particularly repeatedly, has been associated with poor mental health, including posttraumatic stress disorder (PTSD) and major depression, with greater exposures to traumatic events increasing the likelihood of PTSD (*10*,*11*). Exposure to ACEs, which includes polyvictimization (i.e., exposure to multiple types of violence) is associated with increased risk for short- and long-term mental and physical health problems, including suicide risk, risky sexual behaviors, and substance use disorders, and increased risk for early death (*12*).

Addressing risk and protective factors common to multiple forms of violence and substance use might be an effective and efficient way to prevent violence. Family-based strategies include promoting home environments that support healthy development through parenting skill and relationship programs (https://www.cdc.gov/violenceprevention/communicationresources/pub/technical-packages.html#technicalPackages). Multiple community-level, evidence-based strategies for preventing youth violence include modifying physical environments (e.g., mitigating abandoned housing), engaging youths through street outreach, mentoring programs, and changing community norms (https://www.cdc.gov/violenceprevention/communicationresources/pub/technical-packages.html#technicalPackages).

Schools offer a unique opportunity to help reduce youth violence. Schools have direct contact with approximately 50 million students for at least 6 hours a day over a 13-year period and have a role in promoting social, physical, and intellectual development (https://nces.ed.gov/programs/digest/d20/tables/dt20_103.20.asp?current). School-based violence prevention programs typically focus on skill-building to solve problems nonviolently, conflict resolution, and emotional control. Environmental school strategies include those that increase youths’ feelings of connectedness to the school environment and to school staff and prosocial peers. Youths who report feeling connected to school are less likely to engage in violent behaviors and substance use and are more likely to report positive mental health or well-being (*13*,*14*). CDC’s What Works in Schools approach includes a safe and supportive environments strategy (https://www.cdc.gov/healthyyouth/whatworks/what-works-safe-and-supportive-environments.htm) to help students feel more connected to trusted adults at school and at home. Connectedness is a protective factor that might help prevent or reduce substance use, poor mental health, violence, and suicide.

The community and social context is important for the implementation of violence prevention efforts. For example, across communities and other settings, protective factors include youths’ feeling connected to persons in these settings, and having safe spaces where they can talk with trusted adults might promote healthy development and buffer the potentially negative influence of other risks (*5*). However, building connectedness might be challenging when structural inequities such as racism and discrimination are pervasive, and disadvantaged youths are most at risk for experiencing violence. Knowledge gaps remain about how to best address structural inequities (i.e., discrimination and economic adversity) that drive disparities in violence. Strategies such as tax credits for families with children, safe and affordable housing, paid parental leave, livable wages, and economic support for developmentally appropriate child care might help mitigate certain inequities (https://www.cdc.gov/violenceprevention/communicationresources/pub/technical-packages.html#technicalPackages).

Another important approach to reducing the number of suicides and other types of violent deaths is mitigating access to lethal means among those at risk for harming themselves or others. For example, recent reviews suggest that counseling paired with the provision of a safety device can increase secure storage of firearms and that child access prevention laws have been associated with lower rates of youth firearm self-injury, including suicide (*14*,*15*). Additional research could strengthen and guide programs, policies, and practices for the primary prevention of violence, suicide, and substance use.

## Limitations

General limitations for the YRBS are available in the overview report of this supplement (*6*). The findings in this report are subject to at least three additional limitations. First, the question assessing lifetime prescription opioid misuse refers to prescription pain medicine (e.g., the question provides examples of opioid-containing prescription medications only). However, , if students considered nonopioid prescription pain medications when answering, an overestimation of prescription opioid misuse prevalence might have occurred. Second, the YRBS is a cross-sectional, comprehensive youth health survey. More prospective research on witnessing community violence and gun carrying could explore causal mechanisms, strengthening the evidence for prevention efforts. Finally, the question on witnessing community violence was written as a lifetime question. The item does not indicate when the violent act was witnessed, the relationship to the victim, or the number of times the youth might have witnessed the violence. Other behavior questions examined had differing time frames; for example, marijuana use was asked for the past 30 days, whereas opioid use was lifetime. These differences lend credence to the idea that time-specific data on community violence could help improve data-to-action efforts at state and local levels.

## Conclusion

Community violence and gun carrying are significant concerns for youths in the United States. More efforts are needed to develop, adapt, and implement evidence-based interventions for communities that are disproportionately affected by violence and to strengthen the use of violence-related data for prevention efforts, including raising awareness of the burden of community violence and gun carrying. Strategies that address shared risk and protective factors, including family, school, community, and society, are more likely to prevent not only community violence and firearm-related homicides, but also other forms of violence. Ultimately, creating safer schools and communities is essential for all youths to have the same opportunity for health and well-being.

## Footnotes

* See e.g., 45 C.F.R. part 46.102(l)(2), 21 C.F.R. part 56; 42 U.S.C. §241(d); 5 U.S.C. §552a; 44 U.S.C. §3501 et seq.
